# Nerve Growth Factor Regulates Neurolymphatic Remodeling during Corneal Inflammation and Resolution

**DOI:** 10.1371/journal.pone.0112737

**Published:** 2014-11-10

**Authors:** Darci M. Fink, Alicia L. Connor, Philip M. Kelley, Maria M. Steele, Michael A. Hollingsworth, Richard M. Tempero

**Affiliations:** 1 University of Nebraska Medical Center, Eppley Institute for Research in Cancer and Allied Diseases, 985950 Nebraska Medical Center, Omaha, Nebraska 68198-5950, United States of America; 2 Boys Town National Research Hospital, Department of Genetics, 555 North 30^th^ Street, Omaha, Nebraska 68131, United States of America; 3 Boys Town National Research Hospital, Department of Otolaryngology, 555 North 30^th^ Street, Omaha, Nebraska 68131, United States of America; Singapore Immunology Network, Singapore

## Abstract

The cellular and physiologic mechanisms that regulate the resolution of inflammation remain poorly defined despite their widespread importance in improving inflammatory disease outcomes. We studied the resolution of two cardinal signs of inflammation–pain and swelling–by investigating molecular mechanisms that regulate neural and lymphatic vessel remodeling during the resolution of corneal inflammation. A mouse model of corneal inflammation and wound recovery was developed to study this process *in vivo*. Administration of nerve growth factor (NGF) increased pain sensation and inhibited neural remodeling and lymphatic vessel regression processes during wound recovery. A complementary *in vivo* approach, the corneal micropocket assay, revealed that NGF-laden pellets stimulated lymphangiogenesis and increased protein levels of VEGF-C. Adult human dermal lymphatic endothelial cells did not express canonical NGF receptors TrkA and p75^NTR^ or activate downstream MAPK- or Akt-pathway effectors in the presence of NGF, although NGF treatment increased their migratory and tubulogenesis capacities *in vitro*. Blockade of the VEGF-R2/R3 signaling pathway ablated NGF-mediated lymphangiogenesis *in vivo*. These findings suggest a hierarchical relationship with NGF functioning upstream of the VEGF family members, particularly VEGF-C, to stimulate lymphangiogenesis. Taken together, these studies show that NGF stimulates lymphangiogenesis and that NGF may act as a pathogenic factor that negatively regulates the normal neural and lymphatic vascular remodeling events that accompany wound recovery.

## Introduction

Acute inflammation commonly results in either wound recovery or the development of a chronic inflammatory reaction. The biologic mechanisms that direct these outcomes are not well understood, despite the negative clinical implications of chronic inflammation. The concept that wound recovery and the resolution of inflammation are passive processes has been challenged by evidence that active processes trigger a switch from pro- to anti-inflammatory mediators in the tissue microenvironment that in turn resolve inflammation [1]–[3].

Networks of nerves, blood vasculature and lymphatic endothelia are strikingly similar in their anatomical organization and location. Nerves and vasculature respond to overlapping or complementary environmental cues to direct their developmental patterning and maintenance [4]–[9]. Signals elaborated by cells of one network may influence the behavior of cells in an adjacent network [10]. There is evidence for shared neurovascular guidance pathways in conditions of tissue disruption, such as neurodegenerative disease or malignancy [11]–[14].

We hypothesized that common mechanisms regulated neurolymphatic remodeling during inflammation and wound recovery. A corneal model of initial inflammation, wound recovery, and recurrent inflammation was developed to investigate mechanisms that regulate the structure and function of sensory nerves and lymphatic vessels, as these systems influence pain and swelling during the resolution of inflammation. Accessibility and the neurovascular features unique to the cornea make it an ideal system in which to examine neurolymphatic architecture and function during an inflammatory episode and wound recovery. The cornea is densely innervated [15]–[18], primarily with sensory nerves derived from the ophthalmic branch of the trigeminal nerve [19]. Sympathetic nerves, from the superior cervical ganglion [19], and parasympathetic nerves, from the main and accessory ciliary ganglia [20] are less common. During homeostasis, the corneal vasculature is limited to a peri-corneal limbal arcade. Angiogenesis and lymphangiogenesis from the limbus can be stimulated experimentally using suture-induced inflammation [21], [22]. In this work corneal sutures were used to induce initial inflammation; suture removal stimulated wound recovery; and replacement of corneal sutures induced recurrent inflammation.

Examination of several candidate neurovascular guidance molecules for mRNA expression during initial inflammation, wound recovery, and recurrent inflammation in the cornea revealed that NGF gene expression was tightly correlated with these distinct physiologic states. We therefore examined the contribution of NGF to alterations in lymphatic vasculature and neural structures during wound recovery. We also evaluated pain–a clinically-relevant physiological measurement.

These results reveal the capacity of NGF to inhibit neurolymphatic remodeling and the resolution of pain during wound recovery. We show for the first time that NGF can induce lymphangiogenesis as an upstream driver of a hierarchical signaling pathway in which VEGF family members are downstream effectors. These findings implicate NGF as a pathogenic factor that inhibits important aspects of wound recovery.

## Methods

### Mice

All experimental procedures were approved by the Institutional Animal Care and Use Committees of Boys Town National Research Hospital and the University of Nebraska Medical Center in accordance with NIH guidelines. Experiments were carried out in six- to ten-week-old female 129S2/SvPasCrl mice purchased from Charles River Laboratories (Wilmington, MA).

### Corneal Surgical Procedures

Prior to all surgical procedures, mice were anesthetized by intraperitoneal administration of a mixture of ketamine (100 mg/kg) and xylazine (10 mg/kg). Mice were euthanized by ketamine/xylazine overdose or by CO_2_ asphyxiation and cervical dislocation.

The corneal model of initial inflammation, wound recovery, and recurrent inflammation was described previously, as were the injection techniques for VEGF-R2/R3 decoy receptors [22].

#### Administration of NGF during Wound Recovery

2.5S NGF (B.5017, Harlan Laboratories, Indianapolis, IN) was resuspended at 100 µg/mL in PBS, pH 7.4. Approximately 10 µL of suspension was delivered per subconjunctival injection with a 33 gauge needle.

#### Micropellet Preparation and Micropocket Assay Procedures

A 4 mm^2^ nylon mesh was used to mold a mixture of 12% (w/w) poly(2-hydroxyethyl methacrylate) (192066-10G, Sigma-Aldrich, St. Louis, MO) solution in 95% ethanol, sucrose octasulfate-aluminum complex (S0652-1G, Sigma-Aldrich), and cytokine into micropellets measuring approximately 305 µm×305 µm×460 µm. Experimental micropellets contained approximately 200 ng of carrier-free recombinant mouse β-NGF (1156-NG-100/CF, R&D Systems), recombinant human VEGF-C, CF (2179-VC-025/CF, R&D Systems), or PBS only. A 20 or 27 gauge needle was used to create a pocket in the center of the cornea, and a fine forceps was used to place a pellet under the corneal flap.

### Immunofluorescence Imaging of Whole Mount Corneas and Axial Sections and Data Collection

#### Dissection and Staining

Globes were enucleated and fixed in 1% PFA in PBS, pH 7.4, for one hour. Corneas were dissected out and either hemi-sectioned or slit at each quadrant with a small incision. Fixation was repeated for one hour. Corneas were blocked and permeabilized in a sterile-filtered PBS, pH 7.4, solution containing 5.2% BSA, 0.3% Triton X-100, and 0.2% NaN_3_ (blocking solution) for one hour shaking at room temperature. Primary antibodies were diluted in blocking solution and applied to corneas overnight shaking at room temperature. Corneas were washed in a sterile-filtered PBS, pH 7.4, solution containing 0.2% BSA, 0.3% Triton X-100, and 0.2% NaN_3_ (wash buffer) three times for one hour shaking at room temperature. Secondary antibodies were diluted in blocking solution and applied to the corneas overnight shaking at room temperature. After three one-hour washes in wash buffer, corneas were mounted on glass microscope slides with mounting medium containing DAPI and stored at 4°C. Primary antibodies: pRb α-Ms β-III tubulin (ab18207, Abcam, Cambridge, MA), mRt α-Ms Lyve-1 (sc-65647, Santa Cruz Biotechnology, Santa Cruz, CA), ArmHms α-Ms CD-31 (MAB1398Z, EMD Millipore, Billerica, MA), Rt α-Ms phospho-histone H3 (H9908, Sigma-Aldrich, St. Louis, MO). Secondary antibodies: 488 Dky α-Rb IgG (AlexaFluor A21206, Life Technologies, Carlsbad, CA), 488 Dky α-Rt IgG (AlexaFluor A21208, Life Technologies), 549 Dky α-Rt IgG (DyLight 712-505-150, Jackson ImmunoResearch Laboratories, West Grove, PA), 649 Gt α-ArmHms IgG (DyLight 127-495-160, Jackson ImmunoResearch Laboratories).

#### Whole Mount Imaging

Whole mount corneas were visualized on a Zeiss Axio A.1 epifluorescence microscope or a Leica stereoscope. 100x epifluorescence images were obtained using SPOT Advanced software (SPOT Imaging Solutions, Sterling Heights, MI). 32x stereofluorescence images were obtained using Leica Application Suite software (Leica Microsystems, Inc., Buffalo Grove, IL). 200x and 400x z-stacks were obtained using a Zeiss 510META confocal microscope (Carl Zeiss AG, Oberkochen, Germany). Images were compiled and analyzed using ZEN 2009, BioImageXD [23] and ImageJ [24] software packages.

#### Quantification of Corneal Nerve Density

Epifluorescence images were color-inverted and overlaid with a 25,000 pixels^2^ grid with random offset using ImageJ. Portions of the grid lying outside of or overlapping the limbal arcade were excluded from the analysis. β-III tubulin^+^ nerves intersecting grid line segments were manually counted in the X and Y directions. To derive density from counted intersections, raw data were normalized to the number of grid squares counted in each image. Both intact and hemisected whole mount corneas were included in the analysis.

Corneal wound bed nerve density was derived by using ImageJ to superimpose a grid over β-III tubulin immunofluorescence photomicrographs. A line was drawn around each wound bed and the area measured using ImageJ. Nerves intersecting gridlines within the wound bed area were quantified and counts normalized to wound bed area.

#### Quantification of Corneal Nerve Clusters

Corneal nerve clusters were defined as tortuous nerve endings organized in a clustered pattern originating from a single larger nerve and extending in three dimensions. Clusters were identified and counted manually from epifluorescence images of intact and hemisected corneal whole mounts.

#### Quantification of Nerve Density at Micropellet

Epifluorescence or confocal images were overlaid with a 3,000 pixels^2^ grid with random offset using ImageJ. Portions of the grid lying outside of the pellet were excluded from the analysis. β-III tubulin^+^ nerves intersecting grid line segments were manually counted. To derive density from counted intersections, raw data were normalized to the number of grid squares counted in each image.

#### Quantification of Lymphatic Vessel Density and Length

Epi- or stereofluorescence micrographs were imported into ImageJ and overlaid with a 0.02 inches^2^ grid with random offset. To determine lymphatic vessel density, grid squares displaying Lyve-1^+^ fluorescent signal were quantified using the Cell Counter feature of the ImageJ software. Grid squares intersecting the limbal arcade were excluded from the quantification. To quantify lymphatic vessel length, the ImageJ Freehand Line tool was used to trace along the length from origin at the limbus to tip of every-other Lyve-1^+^ lymphatic vessel in stereofluorescence micrographs and the length of each line measured using the ImageJ Measure feature.

#### Quantification of Lymphatic Vessel Fragments

A lymphatic vessel fragment was defined as a group of Lyve-1^+^ lymphatic endothelial cells present in the cornea that was no longer continuous with vessels sprouted from the limbal lymphatic vessel. Following exogenous administration of NGF or PBS during wound recovery, Lyve-1 immunostained corneas were analyzed by epifluorescence microscopy. The number of lymphatic vessel fragments per cornea was counted manually.

#### Quantification of Average Remaining Wound Size

Light stereofluorescence micrographs were imported into ImageJ and the Freehand Line and Measure Area tools were used to trace around each of the four wound beds in each image and to quantify the areas. The area of each wound bed was treated as an individual *n* in further analyses.

### Aesthesiometry

A Luneau Cochet-Bonnet aesthesiometer (Ophthalmic Instrument Co., Inc., Stoughton, MA) was used to measure corneal sensitivity. The nylon monofilament of the aesthesiometer was extended to its full length of 6.0 cm and touched to the corneal surface until first visible bending. Monofilament length was decreased by 0.5 cm increments and touched to the cornea again until a blink response was elicited from the animal. If an animal held its eye closed so as to prevent measurement, we assigned a reading of “7.0”.

### *In Vitro* and Biochemical Assays

#### RNA Isolation, cDNA Library Construction, and qRT-PCR Characterization of Expression of Neurovascular Guidance Genes and NGF Receptors Genes

Total RNA was isolated from mouse cornea, uninvolved human skeletal muscle obtained at rapid autopsy from pancreatic cancer patients, and adult human dermal lymphatic endothelial cells (Lonza, Walkersville, MD) by TRIzol (Life Technologies, Carlsbad, CA) or the RNeasy Mini Kit (Qiagen, Venlo, Netherlands) according to the manufacturers’ instructions. 10 µL linear acrylamide (AM9520, 5 mg/mL, Life Technologies) was added to each corneal sample as a carrier prior to RNA isolation. cDNA libraries were prepared using the SuperScript III First-Strand Synthesis System (18080051, Life Technologies). Mouse qRT-PCR reactions were performed in triplicate and assayed on the Applied BioSystems StepOnePlus Real-Time PCR System (Life Technologies). Expression was normalized to GAPDH. Data was analyzed using the ΔΔC_t_ method. Gene expression levels in unmanipulated samples were set at 1.0. Target gene expression levels in other tissue conditions are expressed as fold change relative to unmanipulated control levels.

TaqMan Gene Expression Assays used for mouse qRT-PCR experiments: GAPDH - 4352339E, NGF - Mm00443039_m1, MMP10 - Mm00444630_m1, IL1-α - Mm00439620_m1, BDNF - Mm04230607_s1, Ntf3 - Mm00435413_s1, Nrp1 - Mm00435379_m1, Nrp2 - Mm00803099_m1, Sema3e - Mm00441305_m1, Plxnd1 - Mm01184367_m1, Ntn1 - Mm00500896_m1, Ntn4 - Mm00480462_m1, Unc5b - Mm00504054_m1, Slit2 - Mm00662153_m1, Robo4 - Mm00452963_m1, Robo1 - Mm00803879_m1, Efnb2 - Mm01215897_m1, Ephb4 - Mm01201157_m1, Notch1 - Mm00435249_m1, Cdk5 - Mm00432447_g1, FGF2- Mm00433287_m1, Vegfa - Mm01281449_m1, Vegfc - Mm00437310_m1, Vegfd - Mm00438963_m1 (Life Technologies, Carlsbad, CA).

Human qRT-PCR reactions were performed in triplicate and analyzed on a BioRad C1000 Thermal Cycler CFX96instrument (Bio-Rad Laboratories, Inc., Hercules, CA).

TaqMan Gene Expression Assays used for human qRT-PCR experiments: TBP - Hs00427521_m1, NTRK1 - Hs01021011_m1, NGFR - Hs00609977_m1 (Life Technologies). Expression was normalized to TBP. Data was analyzed using the ΔΔC_t_ method. Average skeletal muscle expression levels of NTRK1 and NGFR were set at 1.0. Average receptor gene expression levels in LEC samples were expressed as fold change relative to skeletal muscle levels. C_t_ values below the level of detection were designated as 40.0, the upper limit of cycles used in these reactions.

#### Lymphatic Endothelial Cell Culture

Adult human dermal lymphatic endothelial cells (LECs) were purchased from Lonza (Walkersville, MD) and cultured to the company’s specifications. Media used to culture LECs was Endothelial Growth Media-2MV supplemented with recommended growth factors (EGM-2MV). For serum starvation Endothelial Basal Media-2 (EBM-2) was used.

#### NGF and LEC Migration Assays

Lower wells of 24-well (8.0 µm pore membrane) Boyden chambers (BD Biosciences, San Jose, CA) were loaded with 750 µL EGM-2MV media diluted 1∶5 with serum-free EBM-2 and supplemented with increasing doses of recombinant mouse NGF (0, 0.5, 1.0, 5.0 µg/ml). 2.5×10^4^ LECs diluted in EBM-2 were seeded into the upper inserts of the Boyden chamber and incubated for 24 hours. After 24 hour migration, membranes were washed with PBS and non-migratory cells were removed by mechanical force with a cotton-tipped applicator. Cells were then fixed and stained using Differential Quik Staining Kit (Polysciences, Inc., Warrington, PA). Membranes were removed from the inserts and mounted on slides. A Nikon Eclipse 90i microscope at 10x magnification was used to image four representative quadrants of the membrane and the number of migratory LECs was quantified.

#### Treatment of LECs with Cytokines

1.5×10^5^ LECs were seeded into 60×15 mm tissue culture plates and allowed to adhere overnight. Cells were then starved for 16 hours in EBM-2. Following starvation, LECs were treated with either fresh EBM-2 or with EBM-2 containing either 4 µg/ml recombinant NGF or 0.5 µg/ml recombinant VEGF-C for 1, 5, 15, or 30 minutes. Cells were then washed twice with PBS and lysates collected with a radioimmunoprecipitation assay (RIPA) lysis buffer (150 mM sodium chloride, 1% IGEPAL, 0.5% sodium deoxycholate, 0.1% SDS, 50 mM Tris buffer) containing a Roche mini PMSF tablet and phosphatase inhibitors sodium fluoride, sodium pyrophosphate, and sodium orthovanadate all at 1 mM.

#### Immunoblotting

Lysates were prepared in RIPA buffer or RayBiotech Cell Lysis Buffer (RayBiotech, Inc., Norcross, GA) and were incubated with 4X SDS sample buffer and 2-mercaptoethanol and run on NuPAGE 10% Bis-Tris gels at 150 V for 75 minutes using the XCell SureLock Mini Cell electrophoresis system (Life Technologies, Carlsbad, CA). Transfer was accomplished using the Mini Trans-Blot Electrophoretic Transfer Cell (BioRad Laboratories, Inc., Hercules, CA) at 100 V for 75 minutes onto Immobilon-FL or Immobilon-P PVDF membranes (EMD Millipore, Billerica, MA). Blots were blocked overnight shaking at 4°C in 5% BSA in PBS with 1X Halt Protease & Phosphatase Inhibitor Cocktail and EDTA (Thermo Scientific, Rockford, IL) or in 5% non-fat dehydrated milk (NFDM) in TBS containing 0.1% Tween-20. Primary antibodies were incubated in 0.1% Tween in PBS (PBS-T) +2.5% BSA or in TBS-T+5% NFDM for 1 to 3.5 hours shaking at room temperature. Blots were washed three times for 15 minutes in PBS-T or TBS-T. Secondary antibodies were incubated in PBS-T+2.5% BSA or TBS-T+5% NFDM for 45 minutes to one hour shaking at room temperature. Blots were washed three times for 15 minutes in PBS-T+0.01% SDS or in TBS-T followed by a one minute wash in ddH_2_O. Blots were imaged using the LI-COR Odyssey fluorescence imaging system and software (Lincoln, NE) or were incubated with chemiluminescence reagents and x-ray film developed with a Kodak X-OMAT 2000 processor.

#### Tubulogenesis Assays

Wells of a 96-well plate were coated with Matrigel Basement Membrane Matrix (BD Biosciences, Bedford, MA) according to manufacturer’s specifications for growth in a three dimensional matrix. LECs were incubated with increasing doses of recombinant human NGF, VEGF-C, or a combination of NGF and VEGF-C diluted in EBM-2 as indicated for 30 minutes at 4°C and then added to Matrigel-coated wells (1.2×10^4^ LECs/well). After four hours, LECs had formed tube-like networks and phase contrast images were collected. Tubulogenesis was quantified by counting the number of tubes per image. Each treatment was performed in triplicate.

#### NGF ELISA

Levels of NGF protein in unmanipulated and inflamed corneas were assayed by ELISA using the Mouse NGF/β-NGF ELISA Kit (EK0470, Boster Biotechnology Co., Ltd., Pleasanton, CA) according to the manufacturer’s instructions. Individual corneal lysates were prepared in RayBiotech Cell Lysis Buffer (RayBiotech, Inc., Norcross, GA), homogenized with a plastic pestle homogenizer, and cleared by centrifugation. Each sample was divided 90/10 into two wells of the ELISA plate and diluted accordingly with sample buffer. Right corneas from three animals made up each group. OD readings were measured in triplicate using a Titertek Multiskan PLUS plate reader.

#### VEGF-A Protein Quantification

Levels of VEGF-A protein were quantified in corneas bearing either a PBS or NGF pellet. We performed a corneal pocket assay and prepared lysates as described above. Lysates from two corneas were combined to make up each sample. Six corneas comprised each group. VEGF-A content was assayed using the Quantibody Mouse Cytokine Array 1 (RayBiotech, Inc., Norcross, GA). The array was scanned using the GenePix 4000B (Molecular Devices (Axon Instruments), Silicon Valley, CA) and data was collected using the GenePix Pro software at several PMT values ranging from 540 to 790 gain. The PMT gain 590 scan generated the best VEGF-A standard curve and data from this scan was analyzed using the Q-Analyzer Software for QAM-CYT-1 (RayBiotech, Inc.).

### Statistical Analysis

Data were analyzed with GraphPad Prism 5 software (GraphPad Software, Inc., La Jolla, CA) using Student’s T-Test or One-Way ANOVA with Dunnett’s Multiple Comparison Post-Test or Bonferroni Post-Test. An asterisk (*) denotes the control group. A bracket between the control and experimental groups indicates statistical significance with *p*<0.05. In cases comparing two experimental groups, a bracket between these groups indicates statistical significance with *p*<0.05.

## Results

### Characterization of neural and lymphatic remodeling in a corneal model of inflammation and resolution

**Figure 1:**
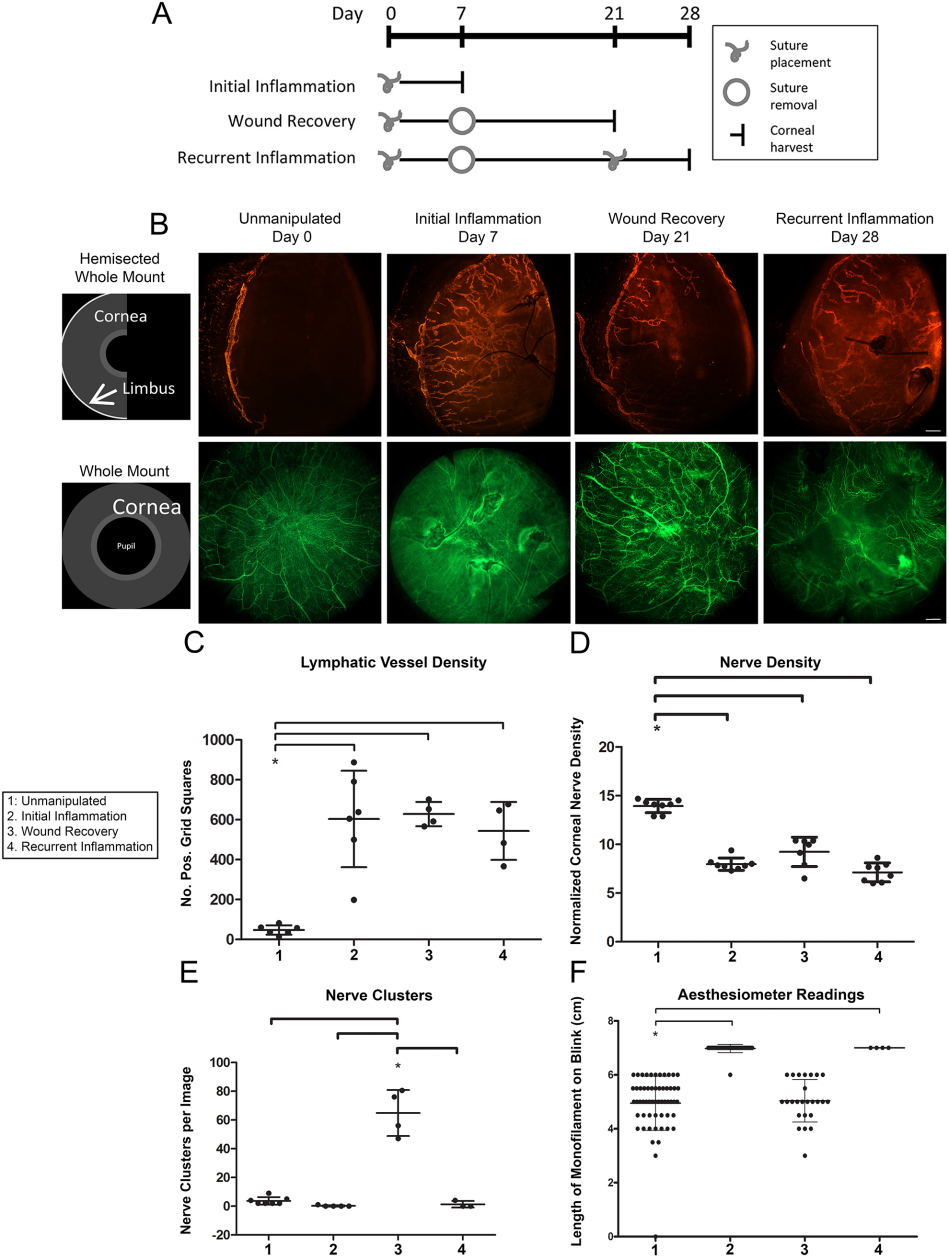
Corneal model of initial inflammation, wound recovery, and recurrent inflammation. *A*. Schematic depicts timing of corneal suture placement and removal to induce four distinct tissue microenvironmental conditions: healthy unmanipulated, initial inflammation, wound recovery, and recurrent inflammation. *B.* Schematics illustrate two corneal mounting styles: hemisected whole mount and whole mount. Images are 100x epifluorescence photomicrographs of corneas immunostained for Lyve-1 (orange) and β-III tubulin (green) and harvested at the indicated time points. *Top* panel shows Lyve-1^+^ lymphatic vessels. *Bottom* panel shows β-III tubulin^+^ corneal nerves. Scale bars are 100 µm. *C.* Lymphatic vessel density was quantified from images like those shown in (*B*). *D.* Nerve density was quantified from images like those shown in (*B*). *E.* Corneal nerve clusters were quantified in wound-recovered tissue. *F.* A Cochet-Bonnet corneal aesthesiometer was used to measure corneal sensitivity.

We characterized structural and architectural changes in corneal lymphatic vessels and nerves through four distinct physiological conditions: healthy unmanipulated cornea, initial inflammation, wound recovery, and recurrent inflammation (**Figure 1A**). In this model system, inflammation was induced by placement of a suture in each quadrant of the mouse cornea. Suture removal stimulated wound recovery. Recurrent inflammation was induced by placement of a second set of four sutures. DAPI-stained corneal axial sections demonstrated gross thickening of the visibly-wounded cornea during both initial and recurrent inflammation (**Figure S1A**). Wound-recovered cornea displayed an intact epithelium and decreased to normal thickness. Levels of interleukin-1α (IL-1α) and matrixmetalloproteinase-10 (MMP-10) messenger RNA by qRT-PCR were consistent with inflammation and wound recovery. IL-1α expression was approximately six-fold higher and MMP-10 eleven-fold higher during initial inflammation compared to unmanipulated controls. During wound recovery, levels of IL-1α and MMP-10 decreased, and levels increased about five-fold with recurrent inflammation (**Figure S1B**).

The lymphatic vessel remodeling that occurs in this system has been previously described [22]. We detected inflammatory lymphangiogenesis associated with initial inflammation, lymphatic vessel regression associated with wound recovery, and an accelerated lymphatic vessel response termed lymphatic vessel memory associated with recurrent inflammation (**Figure 1B, C**).

We uncovered novel neuroremodeling events as we characterized the distinct tissue microenvironments generated in this model system. Detection of nerves by β-III tubulin immunofluorescence revealed three distinct neural phenotypes corresponding to unmanipulated, inflamed, and wound-recovered tissue states, respectively (**Figure 1B**). Unmanipulated control corneas exhibited a very dense plexus of fine nerves organized in a regular swirled distribution present just below the epithelium. These sub-epithelial nerves stemmed from thicker branched nerves lying deep within the corneal stroma and terminated in the epithelium. During initial inflammation, the regular swirled pattern of sub-epithelial nerves was disrupted. These nerves appeared grossly thickened and were present at a relatively low density. Nerves tracked toward sites of injury, often exhibiting 90 degree bends and growing around and through suture knots. Contrary to what we anticipated, nerves in wound-recovered tissue did not re-adopt their pre-morbid swirled morphology. Instead, nerves displayed a novel phenotype characterized by their termination in tight tortuous groups, which we have termed clusters. Quantification of these clusters showed their near-exclusive presence in wound-recovered cornea (**Figure 1E**). Recurrent inflammation resulted in a nerve phenotype very similar to that of initial inflammation with low nerve density and thickened nerves turning toward and knotting around sutures (**Figure 1B, D**).

Given the different nerve morphologies observed, we investigated the possibility that sensory nerve function was consequentially altered. A Cochet-Bonnet aesthesiometer was used to quantify corneal sensitivity and revealed that inflamed corneas were significantly more sensitive than unmanipulated or wound-recovered corneas (**Figure 1F**). This result suggested a connection between pain and the specific neuroremodeling events that accompany inflammation–both initial and recurrent. Although the morphology of nerves in wound-recovered tissue was greatly altered compared to normal cornea, nerves in wound-recovered tissue transmitted sensitivity signals at the same level as normal healthy nerves; only inflamed tissue was more sensitive.

### Expression profiling of NGF and other neurovascular guidance family members

**Figure 2:**
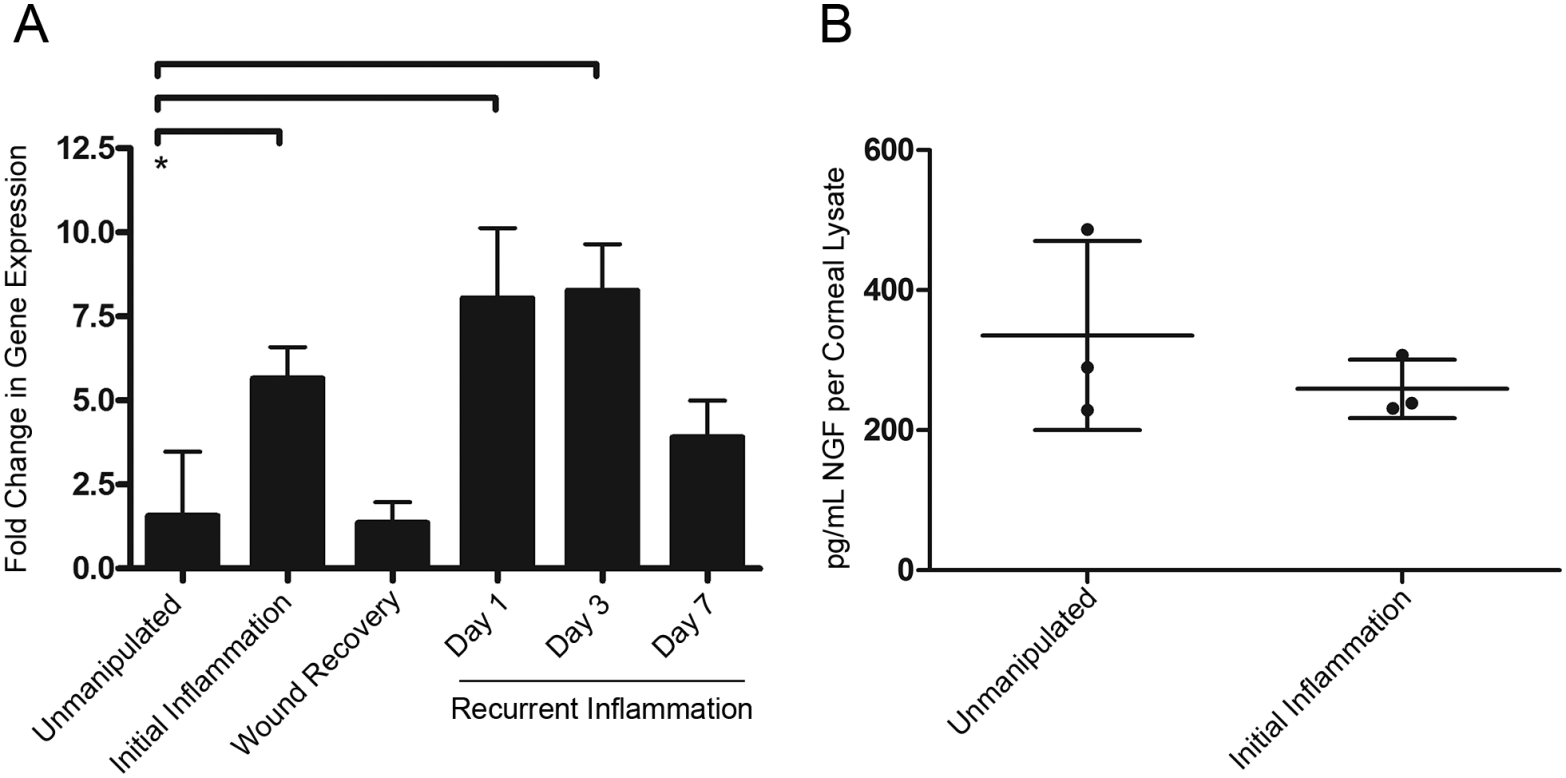
NGF mRNA and protein expression during inflammation and wound recovery. *A.* NGF expression (mRNA) in corneas was evaluated by qRT-PCR in healthy unmanipulated cornea and during initial inflammation, wound recovery, and days one, three, and seven of recurrent inflammation. *B.* NGF protein expression in corneal lysates was evaluated by ELISA.

The dramatic changes in neurolymphatic anatomy during inflammation and wound recovery led us to profile expression of several families of neurovascular guidance genes. RNA was extracted from corneas in the following conditions: unmanipulated, initial inflammation, wound recovery, and one-, three-, and seven-day recurrent inflammation. We sampled the recurrent inflammation condition at three time points in order to better understand the kinetics of a second inflammatory response. Of all of the molecules studied, we observed the most dynamic changes in the expression of NGF (**Figure 2A**). NGF mRNA levels increased with inflammation about six-fold over control and decreased during wound recovery. In recurrent inflammation, NGF levels were increased about seven-fold over control at days one and three, with a more modest increase of approximately four-fold observed at day seven. The robust and rapid increase in NGF mRNA suggested that its expression was correlated with inflammation and wound recovery. We examined the expression of several other neurovascular guidance molecules including two other members of the neurotrophin family: brain-derived neurotrophic factor (*Bdnf*) and neurotrophin-3 (*Ntf3*); four members of the semaphorin/plexin-neuropilin signaling pathway: neuropilin-1 (*Nrp1*), neuropilin-2 (*Nrp2*), semaphorin 3e (*Sema3e*), and plexin-d1 (*Plxnd1*); three members of the netrin/unc family: netrin-1 (*Ntn1*), netrin-4 (*Ntn4*), and unc-5 homolog B (*Unc5b*); three members of the slit/robo family: slit-2 (*Slit2*), roundabout-4 (*Robo4*), and roundabout-1 (*Robo1*); two members of the ephrin/eph family: ephrin-b2 (*Efnb2*), and Eph receptor B4 (*Ephb4*); as well as notch-1 (*Notch1*), cyclin-dependent kinase 5 (*Cdk5*), and fibroblast growth factor 2 (*Fgf2*) (**Figure S2**). Among these, members of the Netrin/Unc and Ephrin/Eph signaling axes were consistently downregulated during inflammation. We did not detect consistent patterns of changes in expression of other neurovascular guidance gene families.

We performed an ELISA for NGF in unmanipulated and initial inflammation corneal lysates to determine if NGF protein levels were upregulated during initial inflammation. The results showed that NGF protein levels did not change with inflammation (**Figure 2B**). These dramatic differences in transcriptional and translational regulation of NGF expression led us to further characterize the role of NGF in neurolymphatic remodeling during inflammation and wound recovery.

### Effects of adding exogenous NGF through wound recovery

We sought to investigate the effect of adding exogenous NGF during wound recovery. It was necessary to first establish the baseline kinetics of neural and lymphatic remodeling during wound recovery, a process that involves the resolution of inflammation. A wound recovery time course experiment was performed in which sutures were removed and corneas were harvested at several time points throughout the course of an extended 21-day wound recovery (**Figure S3A**). Concomitantly, corneal sensitivity was evaluated by aesthesiometry as inflammation resolved. We found that lymphatic vessel density decreased gradually throughout the wound recovery period, while nerve density remained at a constant and relatively low level as compared to controls from previous experiments (**Figure S3B**). Nerve distribution and morphology showed changes throughout the time course. Nerves in early wound recovery (days one through four) displayed features similar to those observed in initial inflammation, *i.e.* tracking toward sites of injury, low density, and overall thickening. Nerves at these time points were also very densely associated with healing wound beds. By day seven of wound recovery, nerves no longer tracked toward wound beds, but rather appeared as loose clusters or individually following tortuous paths. Nerve thickness decreased, approaching that seen in the normal condition. By day ten, the dense networks at wound beds were no longer evident, and clusters were the predominant nerve phenotype visible within the corneal tissue. These clusters persisted through day twenty-one. Assessment of corneal sensitivity by aesthesiometry revealed a steady decrease in the corneal hypersensitivity created by inflammation with a return to normal levels by day seven of wound recovery (**Figure S3C**).

**Figure 3:**
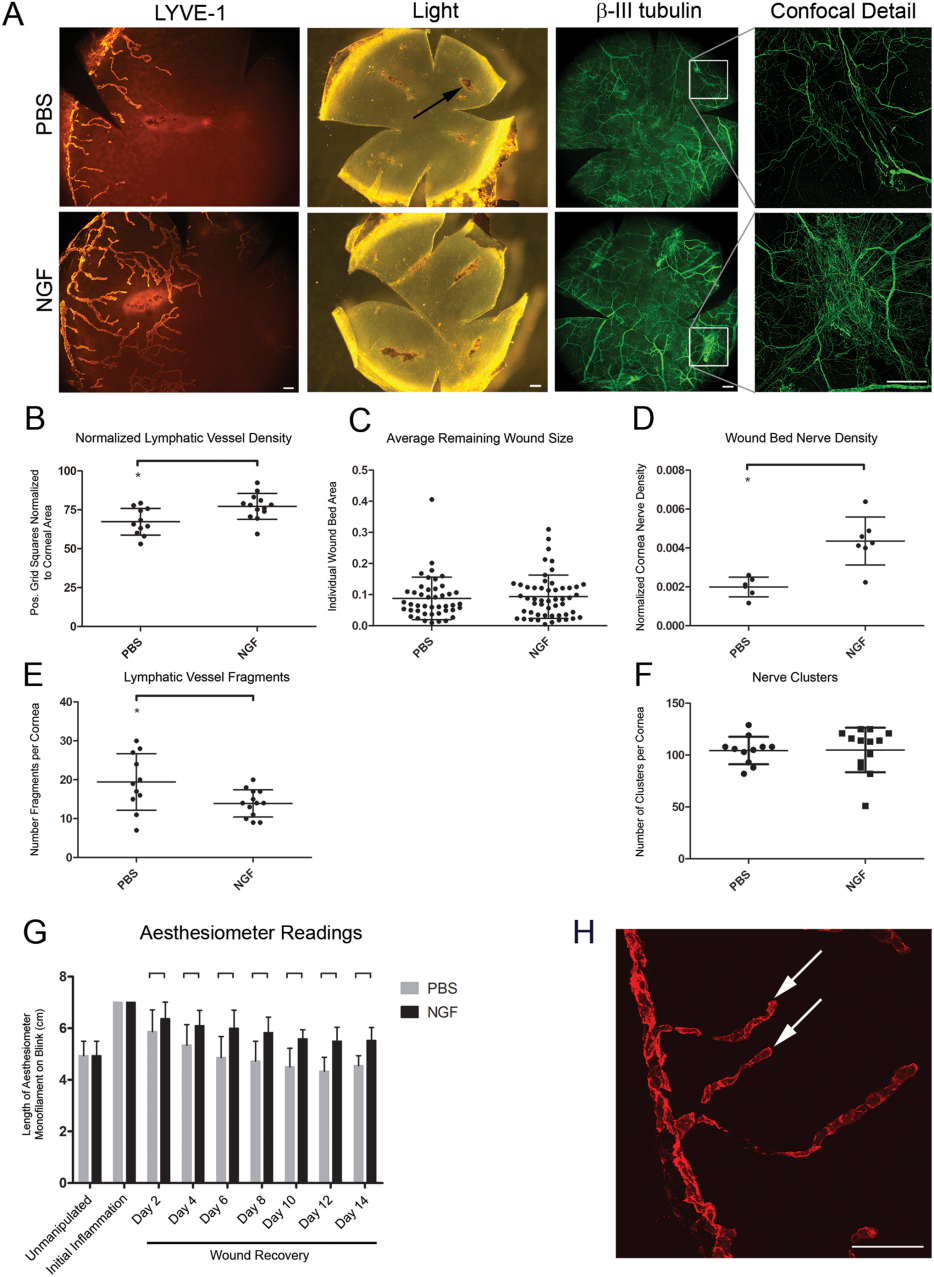
Effects of NGF administration during wound recovery on lymphatic vessel regression, nerve density at corneal wounds, and corneal sensitivity. After removing sutures to stimulate wound recovery, mouse β-NGF or PBS control was injected subconjunctivally every other day for two weeks. Corneas were harvested, immunostained for Lyve-1 and β-III tubulin, and analyzed by whole mount microscopy. *A*. Epifluorescence images of Lyve-1^+^ lymphatic vessels (*left panel,* scale bar = 100 µm, 100x), bright field images showing remaining wound beds at sites of suture placement (*center panel,* scale bar = 200 µm, 32x), epifluorescence images of β-III tubulin^+^ nerves (*right panel,* scale bar = 200 µm, 32x). Expanded detail shows confocal analysis of β-III tubulin^+^ nerves at wound beds (*offset panel far right,* scale bar = 100 µm, 200x). *B*. Analysis of effects of exogenous NGF administration on corneal lymphatic vessel density during wound recovery from images like those in (*A*). *C*. Quantification of average remaining wound size following administration of NGF or PBS. *D*. Nerve density at remaining wound beds quantified from confocal immunofluorescence images. *E.* Quantification of lymphatic vessel fragments discontinuous with limbal lymphatic vessel. *F.* Quantification of nerve clusters in wound-recovered cornea following administration of NGF or PBS. *G*. Measurements of corneal sensitivity throughout wound recovery period with administration of NGF or PBS. *H*. 200x confocal immunofluorescence micrograph showing lymphatic vessel fragments (*arrowheads*). Scale bar = 100 µm.

This better understanding of the dynamic anatomical and physiological events that accompany a period of wound recovery allowed us to test the effects of NGF administration on specific features of wound recovery. Following suture removal to induce wound recovery, mouse β-NGF or PBS control was administered subconjunctivally every other day for fourteen days with commensurate aesthesiometer readings. Lymphatic vessel density remained significantly higher in mice treated with NGF (**Figure 3A, B**), and there was a significant reduction in lymphatic vessel fragmentation (**Figure 3A, E**), a hallmark of normal lymphatic regression during wound recovery (**Figure 3** **H**). We measured remaining corneal ulcers from control and NGF-treated corneas and found no difference in average remaining wound size with NGF administration (**Figure 3A, C**). While nerves did form clusters under both treatment conditions (**Figure 3A, F**), mice treated with NGF showed nerves organized in dense mesh-like networks that encompassed the wound bed (**Figure 3A, D**). NGF-treated corneas were significantly more sensitive than PBS controls at days two through fourteen of wound recovery (**Figure 3G**).

### NGF stimulates lymphangiogenesis

**Figure 4:**
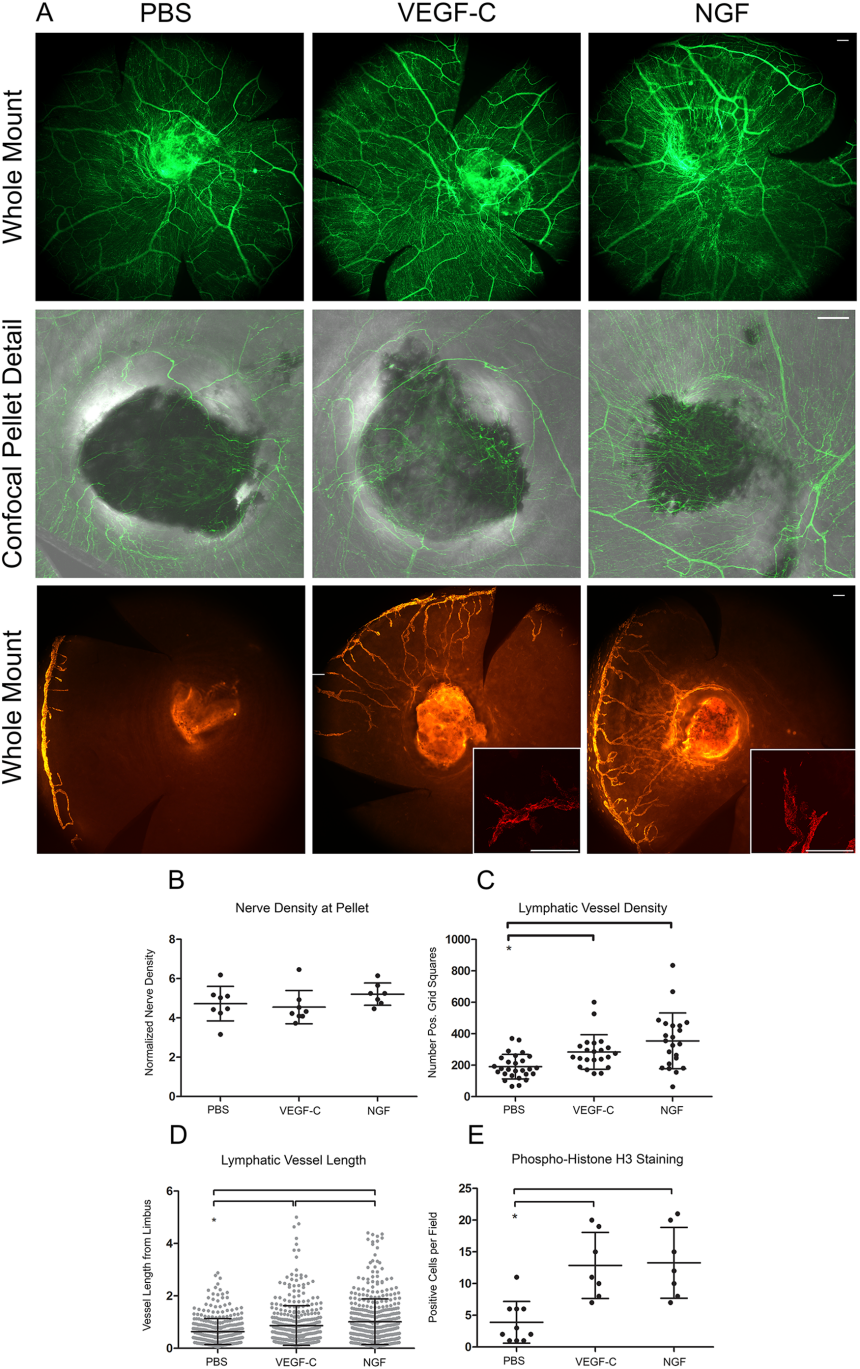
NGF induces lymphangiogenesis. Micropellets loaded with PBS, VEGF-C, or NGF were positioned in a corneal micropocket. Corneas were harvested seven days after pellet implantation, immunostained for Lyve-1, β-III tubulin, and phosphorylated histone H3 and analyzed by whole mount and confocal microscopy. *A*. Epifluorescence images of β-III tubulin^+^ corneal nerves (*top panel,* scale bar = 100 µm, 100x), confocal detail images of β-III tubulin^+^ nerves at micropellets embedded in cornea (*middle panel,* scale bar = 100 µm, 100x), epifluorescence images of Lyve-1^+^ lymphatic vessels (*bottom panel,* scale bar = 100 µm, 100x), and confocal detail images of Lyve-1^+^ lymphatic vessel tip cells (*bottom panel, inset,* scale bar = 100 µm, 200x). *B.* Quantification of corneal nerve density at micropellets loaded with PBS, VEGF-C, or NGF from confocal images like those shown in (*A*). *C*. Quantification of lymphatic vessel density in response to cytokine micropellets from images such as those shown in (*A*). *D.* Quantification of length of lymphatic vessel outgrowth from limbus. *E*. Proliferation of Lyve-1^+^ lymphatic endothelial cells as quantified by positive staining for phosphorylated histone H3.

Given the inhibition of lymphatic vessel regression by NGF, we sought to determine if NGF was capable of inducing lymphangiogenesis in the cornea. A corneal micropocket assay was used to test this hypothesis. Micropellets loaded with PBS control, VEGF-C, or NGF were placed in a corneal micropocket for seven days, after which corneas were harvested and analyzed using immunofluorescence microscopy (**Figure 4A**). β-III tubulin staining showed corneal nerves tracking toward pellets in all three conditions. Nerve density did not change at pellets loaded with cytokine (**Figure 4A, B**). VEGF-C and NGF stimulated lymphangiogenesis (**Figure 4A**). Measurement of lymphatic vessel density (**Figure 4C**) and length (**Figure 4D**) confirmed that VEGF-C and NGF significantly increased lymphangiogenesis over PBS control. NGF-induced lymphatic vessels were significantly longer than those induced by VEGF-C (**Figure 4D**). Analysis of lymphatic endothelial cell proliferation in newly synthesized lymphatic vessels by quantification of phosphorylated histone H3 staining in Lyve-1^+^ cells showed an increased number of proliferation events in the presence of NGF and VEGF-C (**Figure 4E**).

**Figure 5:**
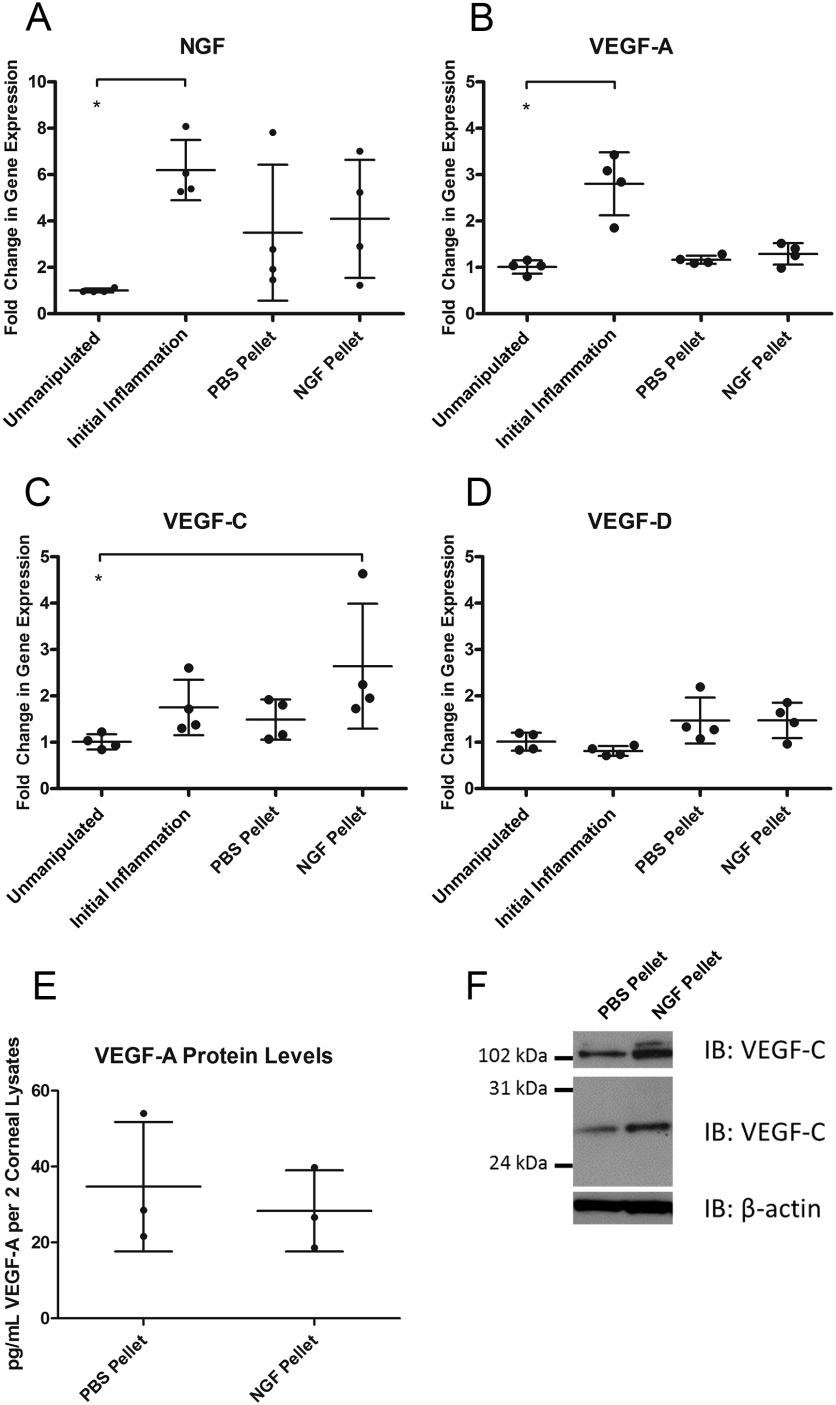
mRNA and protein expression profiling of lymphangiogenic cytokines. Conditions of initial inflammation, PBS pellet, or NGF pellet were created by placement of sutures or appropriate pellet as described. *A.* NGF gene expression from corneal RNA from unmanipulated, initial inflammation, PBS pellet, and NGF pellet conditions. *B*. VEGF-A gene expression. *C*. VEGF-C gene expression. *D*. VEGF-D gene expression. *E.* VEGF-A protein quantification from cytokine array from corneas with PBS pellet or NGF pellet. *F*. VEGF-C protein quantification by western blot from corneas with PBS pellet or NGF pellet. β-actin was included as a loading control.

We also examined the gene expression of NGF and known lymphangiogenic cytokines VEGF-A, -C, and -D in corneas with a PBS or NGF pocket as well as in the unmanipulated and initial inflammation conditions. NGF gene expression increased during initial inflammation and did not change with PBS or NGF pellet placement (**Figure 5A**). VEGF-A gene expression displayed the same pattern as NGF with an increase in initial inflammation and no change in the presence of a PBS or NGF pellet (**Figure 5B**). VEGF-C gene expression trended toward an increase during initial inflammation and was significantly higher than the unmanipulated condition in the presence of an NGF pellet (**Figure 5C**). VEGF-D gene expression remained unchanged in all conditions (**Figure 5D**). We also assayed VEGF-A and -C protein levels in corneas with a PBS or NGF pellet. Quantification of VEGF-A protein levels by cytokine microarray showed no change with an NGF pellet (**Figure 5E**). Western blotting detected an increase in three isoforms of VEGF-C in the presence of an NGF pellet (**Figure 5F**). These results show an increase in both VEGF-C mRNA and protein in the presence of an NGF pellet, but not VEGF-A or -D, and suggest that NGF may induce expression of VEGF-C, the canonical lymphangiogenic cytokine, in the cornea.

### *In vitro* interrogation of NGF activity on adult human dermal lymphatic endothelial cells

**Figure 6:**
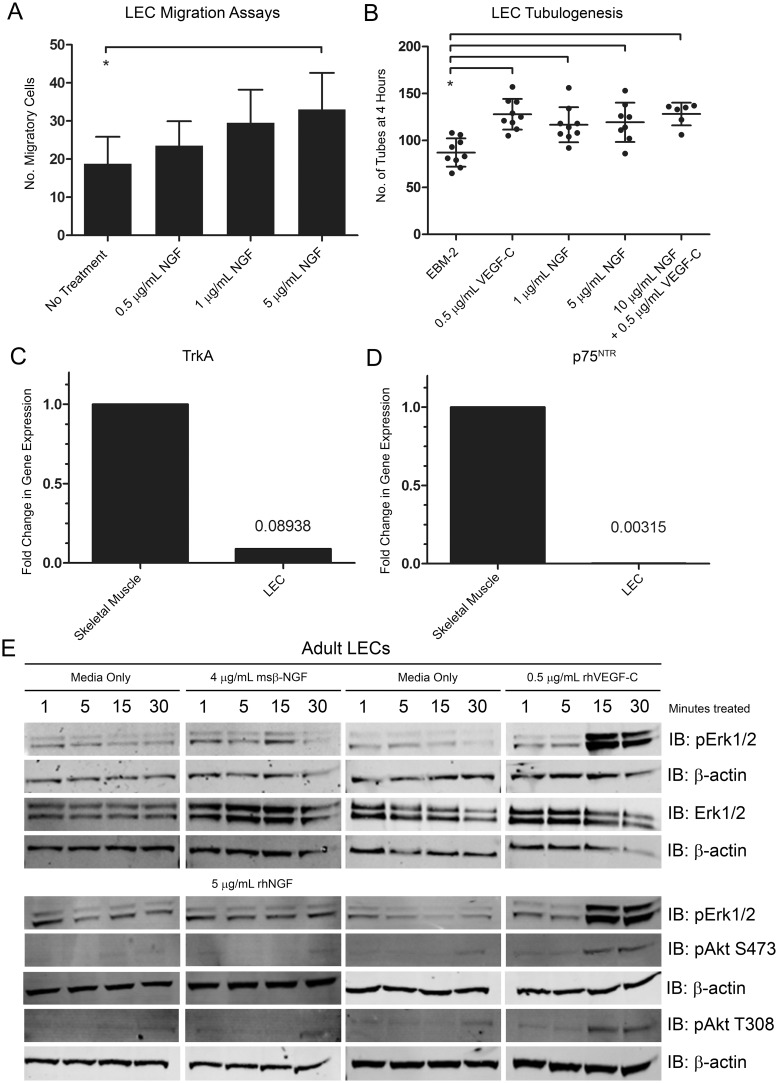
*In vitro* experiments examining the effects of NGF on LECs. *A.* Adult human dermal lymphatic endothelial cells (LECs) were seeded in the upper inserts of Boyden chamber migration plates. NGF was added to the lower wells at the indicated concentrations. Migratory cells were quantified. *B.* Matrigel was placed in tissue culture plate wells. LECs were incubated with the indicated cytokines and then added to wells. Tubes were quantified at four hours. *C.* RNA was extracted from LECs or positive control adult human skeletal muscle and cDNA was synthesized. Gene expression levels of NGF receptor TrkA were quantified by qRT-PCR. *D.* Gene expression levels of p75^NTR^ in human skeletal muscle and LECs. *E.* RIPA lysates were made from LECs treated with the indicated cytokines for 1, 5, 15, or 30 minutes. Western blot analysis was performed for the indicated proteins using β-actin as a loading control.

Considering that NGF stimulated corneal lymphatic vessels and VEGF-C mRNA and protein expression *in vivo*, we investigated whether NGF acted directly on lymphatic endothelial cells (LECs). We examined the potential of NGF as a chemoattractant in Boyden-chamber migration assays. NGF induced a modest but statistically significant increase in migration of LECs (**Figure 6A**). Because of the low numbers of migratory cells in these assays, we postulated that NGF might have activity only on a subpopulation of cells or that VEGF-C might be required for robust migration. We also examined the potential of NGF to stimulate tube formation in LECs. Two concentrations of NGF, positive control VEGF-C, and the two cytokines in combination were equally capable of increasing tubulogenesis in LECs cultured in Matrigel at four hours (**Figure 6B**).

LECs were evaluated for expression of mRNA for canonical NGF receptors TrkA and p75^NTR^ by qRT-PCR. Human skeletal muscle was used as a positive control for expression of these receptors. Gene expression of TrkA was 11.2-fold lower in LECs than in skeletal muscle (**Figure 6C**). Expression of p75^NTR^ was 317.1-fold lower in LECs than in skeletal muscle (**Figure 6D**). These results suggested that these receptors were not expressed on these cells.

We studied the expression of signaling phosphoproteins downstream of receptor tyrosine kinases that are known to induce LEC proliferation and migration. Levels of pErk1/2 and pAkt were examined in LEC lysates treated with NGF or positive-control VEGF-C for 1, 5, 15, or 30 minutes. As expected, VEGF-C induced phosphorylation of pErk1/2 and pAkt at 15 and 30 minutes. NGF treatment did not induce phosphorylation of these downstream effectors (**Figure 6E**).

Taken together, these results suggest that NGF does not act through its canonical receptors or through typical receptor tyrosine kinase signaling pathways on populations of LECs in culture.

### VEGF-R2/R3 decoy receptors block NGF pellet-mediated corneal lymphangiogenesis

A combinatorial experimental approach was taken to investigate the hierarchical nature of NGF/VEGF-C signaling *in vivo.* An initial set of experiments showed that subconjunctival injection of VEGF-R2/R3 decoy receptors during initial inflammation blocked suture-mediated lymphangiogenesis while having no effect on overall corneal nerve density (**Figure S4A, B**). This result confirmed the efficacy of ablating VEGF-R2/R3 signaling on inflammatory lymphangiogenesis and suggested that blocking this signaling mechanism does not affect inflammatory neuroremodeling.

**Figure 7:**
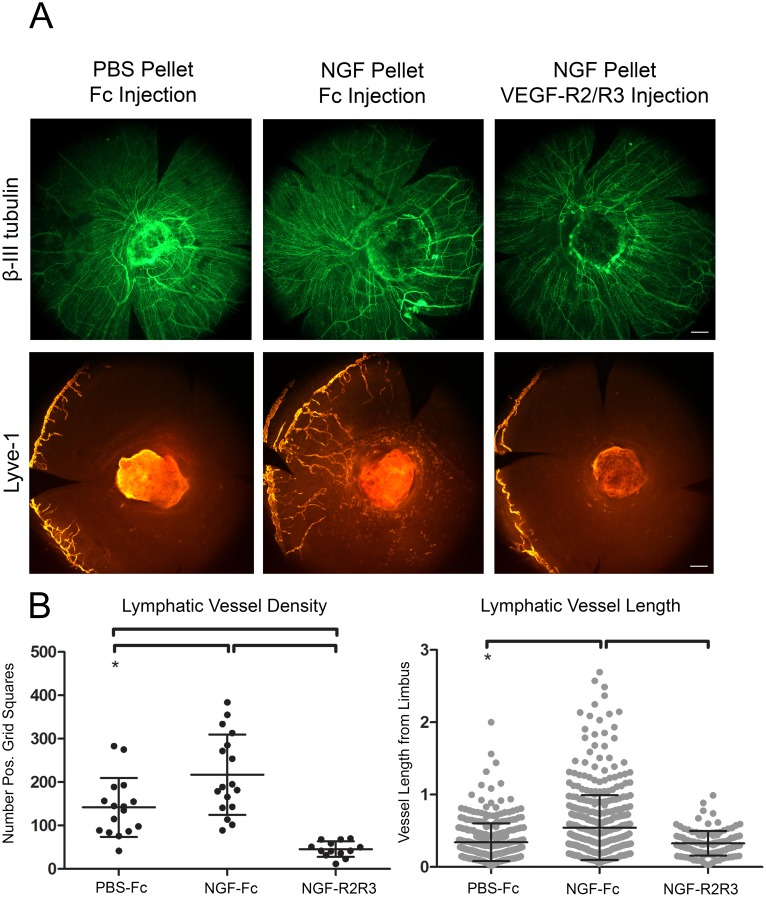
VEGF-R2/R3 decoy receptors ablate NGF pellet-mediated corneal lymphangiogenesis. Micropellets loaded with PBS or NGF were positioned in a corneal micropocket on experimental day zero. Fc control or a solution of VEGF-R2/R3 decoy receptors was administered subconjunctivally on days zero, two, and four. Corneas were harvested on day seven, immunostained for Lyve-1 and β-III tubulin, and analyzed by whole mount microscopy. *A*. 100x epifluorescence images of β-III tubulin^+^ corneal nerves (*top panel*) and Lyve-1^+^ lymphatic vessels (*bottom panel*). Scale bars = 100 µm. *B*. Quantification of lymphatic vessel density (*left panel*) and length of outgrowth from limbus (*right panel*).

We investigated the effects of VEGF-R2/R3 signaling blockade on NGF-mediated lymphangiogenesis. Micropellets loaded with PBS or NGF were placed in the cornea to induce lymphangiogenesis followed by subconjunctival injection of VEGF-R2/R3 decoy receptors or Fc control (**Figure 7A**). PBS control pellet paired with Fc control subconjunctival injection stimulated minimal lymphangiogenesis. NGF-laden pellet and Fc control injection induced robust lymphangiogenesis. NGF-laden pellet coupled with blockade of VEGF-R2/R3 signaling by decoy receptor injection ablated lymphangiogenesis. Lymphatic vessel density was significantly higher in the NGF pellet and Fc injection condition, while the addition of VEGF-R2/R3 decoy receptors to the system significantly reduced lymphatic vessel density and length (**Figure 7B**). These results suggest that the capacity of NGF to induce lymphangiogenesis in the cornea is largely mediated through indirect effects (stimulation of production of VEGF-C) on cell types other than lymphatic endothelial cells.

## Discussion

Our studies investigated the mechanistic basis for the resolution of two of the cardinal features of inflammation–pain and tissue edema–during wound recovery. We characterized the behavior of two classes of tissue microenvironmental structures in the cornea, the nerves and the lymphatic vessels, during episodes of inflammation and wound healing. We show synchronized neurolymphatic remodeling during inflammation and wound recovery that is regulated by NGF and its downstream effectors, including VEGF-C. These studies demonstrate that NGF stimulates lymphangiogenesis and increases pain, and they implicate NGF as a pathogenic factor that inhibits the neural and lymphatic vascular remodeling processes associated with wound recovery.

### Neurolymphatic Remodeling and Wound Recovery

A corneal model of initial inflammation, wound recovery, and recurrent inflammation was used to explore mechanisms that coordinately regulate neurolymphatic remodeling. This study features experimental results from wound recovery and recurrent inflammation conditions, two understudied physiologic states with significant clinical relevance. Histological and biochemical results supported our conclusion that this model represents these physiologic states.

We and others have previously shown that sutures induce lymphangiogenesis from the corneal limbus and that suture removal stimulates wound recovery and lymphatic vessel regression [21]. Suture placement may transect or induce neuropraxia in a subset of corneal nerves. However, the data presented here reveal new aspects of neural and lymphatic responses to inflammation and injury, processes similar to severe clinical corneal infection or direct trauma.

During initial inflammation, we observed synthesis of new Lyve-1^+^ lymphatic vessels and remodeling of β-III tubulin^+^ neural structures to wound-centric tracking with sharp directional turns. Nerves also displayed thickening and a decrease in density, similar to previous observations [25], [26]. We visualized regressed Lyve-1^+^ lymphatic vessels and β-III tubulin^+^ neural clusters during wound recovery. Recurrent inflammation accelerated the development of a lymphatic vessel network [22] and induced neural remodeling, changing nerve clusters to a wound-centric organization. We also detected marked changes in corneal sensitivity that represented a physiological consequence of neural remodeling events. Among several classes of neurovascular guidance molecules, we identified NGF mRNA to have increased expression during inflammation compared to unmanipulated control and wound recovery conditions. Surprisingly, NGF protein levels were similar in control compared to conditions of initial corneal inflammation. Expression of immature or alternately processed biologically active forms of NGF, such as proNGF or other cleavage or post-translationally modified products, may not have been detected by the ELISA we performed.

It is well known that several cell types present in the cornea including epithelium, endothelium, keratocytes, and nerves express NGF and/or the NGF receptors TrkA and p75^NTR^ [27]–[32]. Here we show that NGF inhibited the normal processes of wound recovery by inhibiting lymphatic vessel regression, maintaining high nerve density in wounds, and increasing pain. Interestingly, NGF did not appear to affect epithelial wound closure. Previous studies have described conflicting results related to the effects of manipulating the NGF pathway. Administration of NGF to the cornea has been shown to increase presence of corneal nerves and increase rates of epithelial proliferation [30], [32], [33], improve corneal healing after capsaicin administration and epithelial scraping [34], and increase wound closure [28]. Other studies have shown that NGF has no effect on wound closure [27]. Administration of an NGF blocking antibody has been shown either to slow epithelial wound healing [28] or to have no effect [27]. These different observations may reflect model-to-model variability and possibly the pan-regulatory functions of NGF.

### NGF Induced Lymphangiogenesis

In our studies with micropellets loaded with NGF, we documented the unexpected finding of stimulating new lymphatic vessel growth. We considered the possibility that NGF acted directly on LECs that expressed the canonical receptors for NGF, TrkA and p75^NTR^, or a non-canonical receptor tyrosine kinase. The data did not support this hypothesis, as we did not detect TrkA and p75^NTR^ expression or phosphorylation of two known downstream signaling mediators, Erk and Akt, in LECs treated with NGF. The results of LEC migration and tubulogenesis assays were intriguing and suggested that NGF may bind to a non-canonical receptor and transduce migratory signals *via* effectors other than Erk or Akt. We explored an alternative but non-exclusive hypothesis that NGF stimulated LECs indirectly through effects on other cell types.

Members of the VEGF family have been shown to induce lymphangiogenesis directly by interacting with VEGF receptors expressed by LECs [35]–[38]. We explored this *in vivo* and showed that NGF-laden pellets stimulated expression of VEGF-C mRNA and protein and that lymphangiogenesis stimulated by NGF pellets was dependent upon the VEGF-A and -C signaling axes.

This is the first report describing that NGF stimulates lymphangiogenesis, indirectly *via* VEGF family members. Blocking VEGF-A and -C signaling with decoy receptors ablated both VEGF-C- and NGF-induced lymphangiogenesis and did not affect neural remodeling. NGF pellets have also been shown to induce angiogenesis in rat corneas [39], and other studies have defined a relationship between the VEGF and NGF signaling axes. Production of VEGF-A has been documented in several models of neural development, disease, or injury and has been shown to be stimulated by NGF administration under certain conditions, presenting the possibility that corneal nerves are the source of one or more VEGF family members [40]–[45]. Studies with bevacizumab, which binds VEGF-A [46], [47], provide further support for crosstalk between these two signaling pathways including changes in NGF levels [48]–[50] and nerve activity [47], [51], [52]. The results of the *in vivo* studies presented here support a similar model of NGF and VEGF-C cross regulation to coordinately regulate neural structures and lymphatic vessel growth. These findings are consistent with a model in which NGF functions as an overarching regulatory molecule with primary neural targets and downstream secondary targets regulating lymphangiogenesis *via* VEGF family members.

### Facilitating Wound Recovery

The resolution of inflammation and wound recovery are universally important biological processes. Here we investigated these processes in two basic physiologic systems, the lymphatic vasculature and the nervous system in the cornea. The distribution, density, and functions of the lymphatic vessels and nerves vary considerably from organ to organ presumably to meet the unique physiologic and pathologic challenges presented in specific microenvironments. Whether the operational mechanisms that we have defined in the cornea can be generalized to other organs systems is unclear.

Other preclinical and clinical studies have investigated the contribution of NGF to corneal disease. Consistent with the findings reported here, TrkA^−/−^ mice display decreased innervation of corneal stroma and epithelium and decreased corneal sensitivity [53], and clinical studies have shown that topical NGF treatment generally increased corneal sensitivity [54], [55]. In other clinical studies, topical administration of NGF eye drops has been perceived as generally favorable by observations of decreased healing time and increased epithelial closure in refractive disease [56]–[62], presumably by stimulating epithelial migration [32]. The findings presented here suggest that NGF serves as part of a signaling hierarchy that regulates neurolymphatic remodeling in the cornea, and that clinical strategies to block NGF function may facilitate wound recovery by modulating these processes.

## Supporting Information

Figure S1 **Induction of inflammation, wound recovery, and recurrent inflammation in the mouse cornea.** Corneal surgeries were performed as described in Figure 1A to induce initial inflammation, wound recovery, and recurrent inflammation. Corneas were harvested and stained with DAPI for epifluorescence microscopy analysis or RNA was extracted for gene expression analysis. *A.* Schematic depicts mount style of frozen corneal axial sections. 200x DAPI-stained corneal axial section epifluorescence micrographs. Scale bar = 100 µm. *B.* qRT-PCR results showing levels of gene expression of inflammatory cytokines MMP10 (*left panel*) and IL1-α (*right panel*).
(TIF)

Figure S2 **Changes in neurovascular guidance molecule gene expression during initial inflammation, wound recovery, and recurrent inflammation time course.** Corneal surgeries were performed as described to induce initial inflammation, wound recovery, and a recurrent inflammation time course. RNA was extracted for qRT-PCR analysis of genes representing neurovascular guidance families.
(TIF)

Figure S3 **Wound recovery time course.** Wound recovery was stimulated by suture removal and corneas were harvested at the indicated time points, immunostained for Lyve-1 and β-III tubulin, and analyzed by epifluorescence microscopy. *A.* 100x immunofluorescence micrographs of Lyve-1^+^ lymphatic vessels (*top panel*) and β-III tubulin^+^ nerves (*bottom panel*). Scale bars = 100 µm. *B*. Quantification of corneal nerve density from images like those in (*A*). *C*. Measurements of corneal sensitivity through extended wound recovery time course.
(TIF)

Figure S4 **VEGF-R2/R3 decoy receptor treatment ablates suture-mediated lymphangiogenesis and does not affect neural remodeling.** Corneas were inflamed with four sutures on experimental day zero. Fc control or a solution of VEGF-R2/R3 decoy receptors was administered subconjunctivally on days zero, two, and four. Corneas were harvested on day seven, immunostained for Lyve-1 and β-III tubulin, and analyzed by whole mount epifluorescence microscopy. *A.* 100x immunofluorescence micrographs of Lyve-1^+^ lymphatic vessels (*top panel*) and β-III tubulin^+^ nerves (*bottom panel*). Scale bar = 100 µm. *B*. Quantification of corneal nerve density from images like those in (*A*).
(TIF)
