# Supplementary figures and images for: Nerve Growth Factor Regulates Neurolymphatic Remodeling during Corneal Inflammation and Resolution
Source: PLoS One. 2014 Nov 10;9(11):e112737. doi: 10.1371/journal.pone.0112737 (PMC4226611; doi:10.1371/journal.pone.0112737)

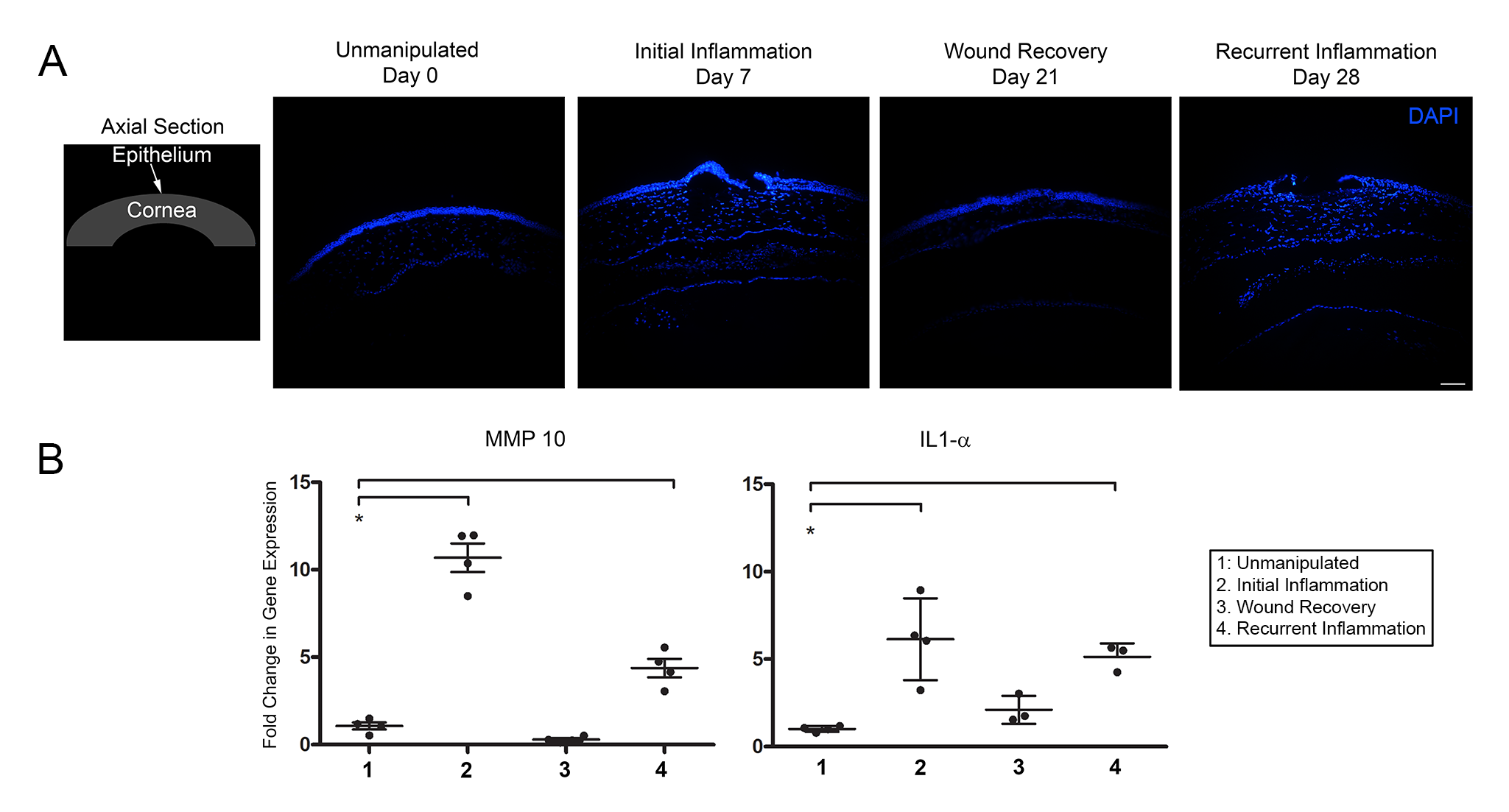

Supplement: Figure S1 — Induction of inflammation, wound recovery, and recurrent inflammation in the mouse cornea. Corneal surgeries were performed as described in Figure 1A to induce initial inflammation, wound recovery, and recurrent inflammation. Corneas were harvested and stained with DAPI for epifluorescence microscopy analysis or RNA was extracted for gene expression analysis. A. Schematic depicts mount style of frozen corneal axial sections. 200x DAPI-stained corneal axial section epifluorescence micrographs. Scale bar = 100 µm. B. qRT-PCR results showing levels of gene expression of inflammatory cytokines MMP10 (left panel) and IL1-α (right panel). (TIF) [file pone.0112737.s001.tif]

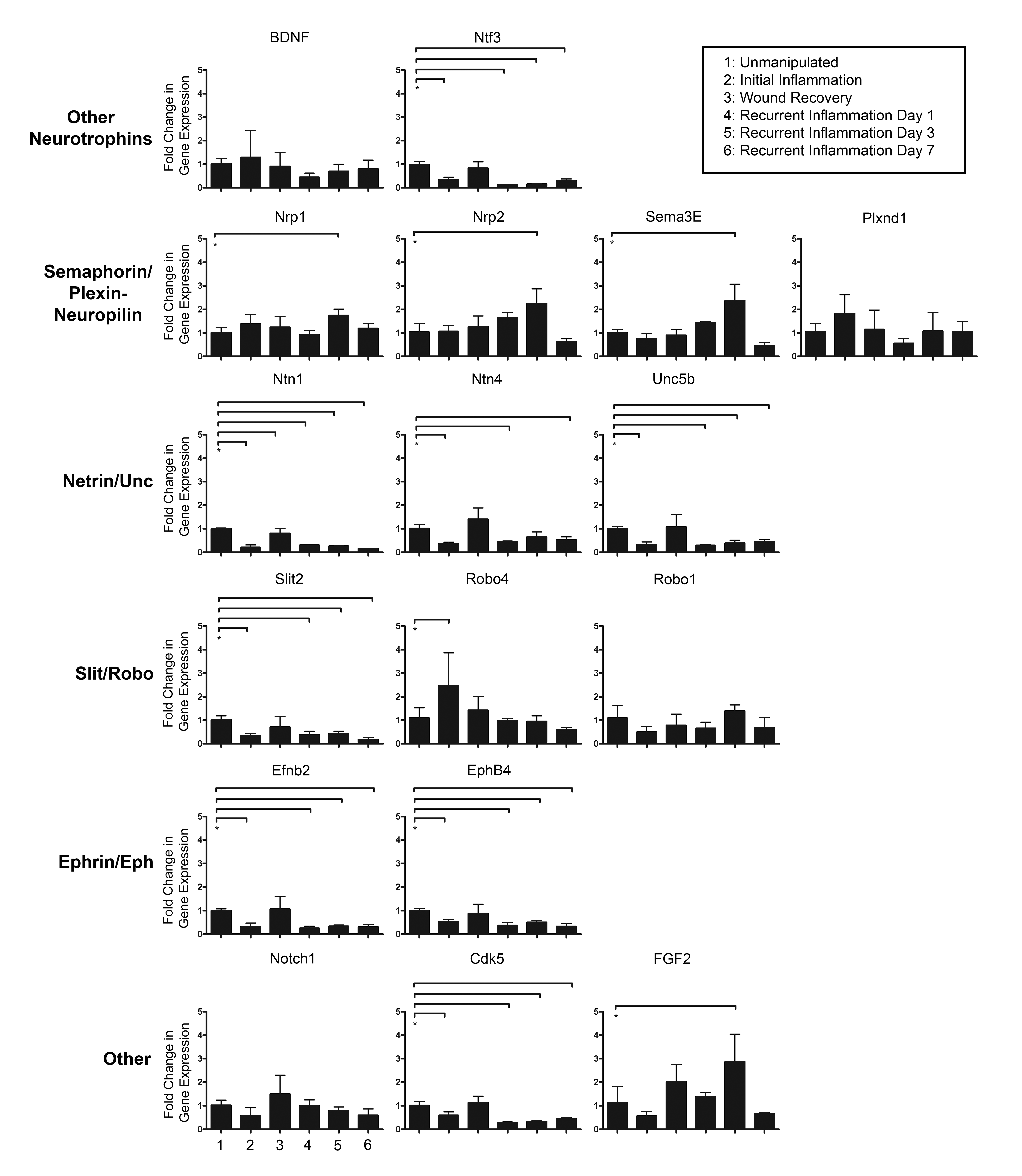

Supplement: Figure S2 — Changes in neurovascular guidance molecule gene expression during initial inflammation, wound recovery, and recurrent inflammation time course. Corneal surgeries were performed as described to induce initial inflammation, wound recovery, and a recurrent inflammation time course. RNA was extracted for qRT-PCR analysis of genes representing neurovascular guidance families. (TIF) [file pone.0112737.s002.tif]

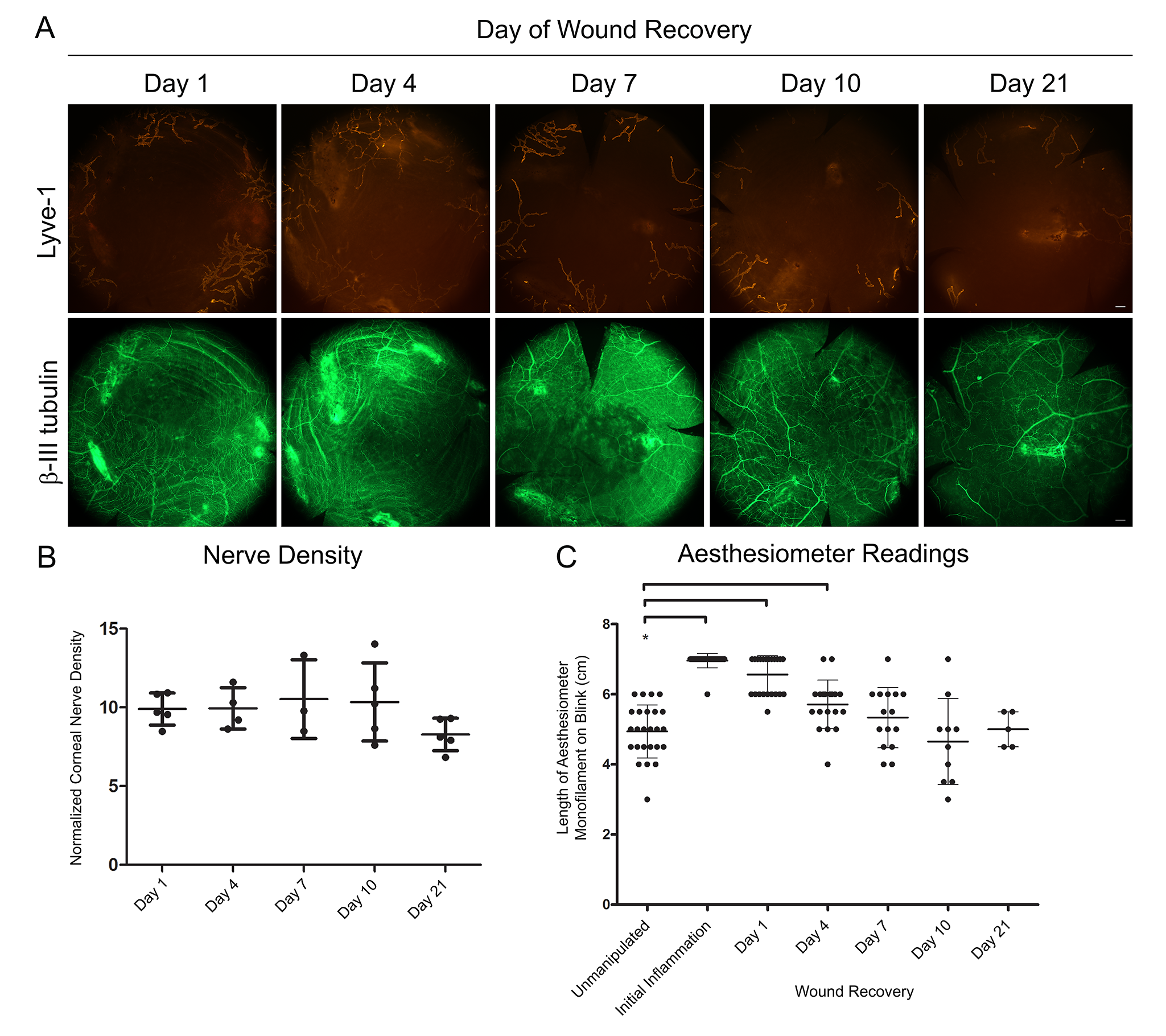

Supplement: Figure S3 — Wound recovery time course. Wound recovery was stimulated by suture removal and corneas were harvested at the indicated time points, immunostained for Lyve-1 and β-III tubulin, and analyzed by epifluorescence microscopy. A. 100x immunofluorescence micrographs of Lyve-1+ lymphatic vessels (top panel) and β-III tubulin+ nerves (bottom panel). Scale bars = 100 µm. B. Quantification of corneal nerve density from images like those in (A). C. Measurements of corneal sensitivity through extended wound recovery time course. (TIF) [file pone.0112737.s003.tif]

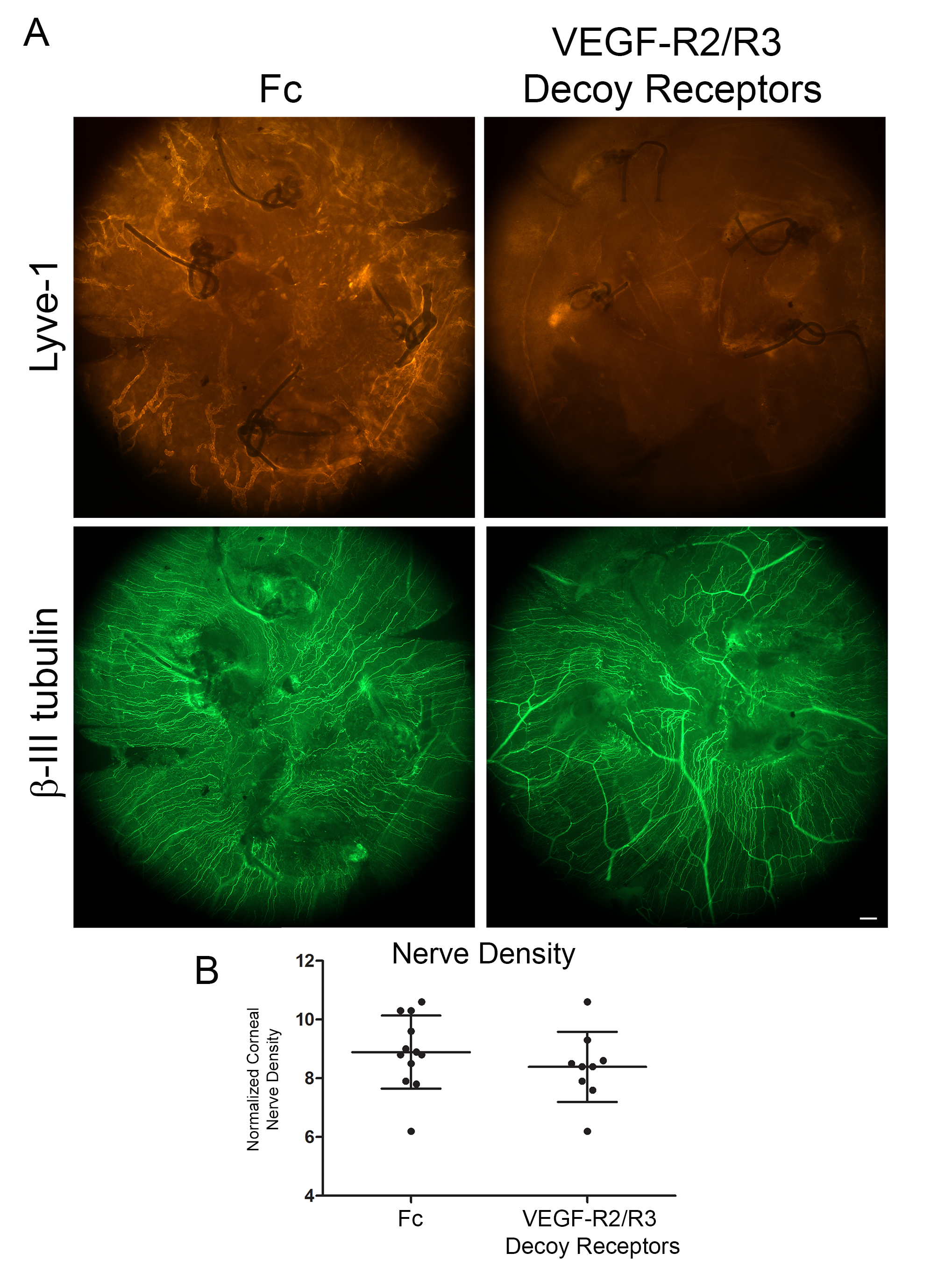

Supplement: Figure S4 — VEGF-R2/R3 decoy receptor treatment ablates suture-mediated lymphangiogenesis and does not affect neural remodeling. Corneas were inflamed with four sutures on experimental day zero. Fc control or a solution of VEGF-R2/R3 decoy receptors was administered subconjunctivally on days zero, two, and four. Corneas were harvested on day seven, immunostained for Lyve-1 and β-III tubulin, and analyzed by whole mount epifluorescence microscopy. A. 100x immunofluorescence micrographs of Lyve-1+ lymphatic vessels (top panel) and β-III tubulin+ nerves (bottom panel). Scale bar = 100 µm. B. Quantification of corneal nerve density from images like those in (A). (TIF) [file pone.0112737.s004.tif]
